# *Vibrio cholerae* O1 in 2 Coastal Villages, Papua New Guinea

**DOI:** 10.3201/eid1701.100993

**Published:** 2011-01

**Authors:** Alexander Rosewell, Rosheila Dagina, Manoj Murhekar, Berry Ropa, Enoch Posanai, Samir R. Dutta, Amy Jennison, Helen Smith, Glen Mola, Anthony Zwi, C. Raina MacIntyre

**Affiliations:** Author affiliations: World Health Organization, Port Moresby, Papua New Guinea (A. Rosewell, M. Murhekar);; National Department of Health, Port Moresby (R. Dagina, B. Ropa, E. Posanai);; Port Moresby General Hospital, Port Moresby (S.R. Dutta);; Reference Microbiology and Molecular Epidemiology Laboratories, Archerfield, Queensland, Australia (A. Jennison, H. Smith);; University of Papua New Guinea, Port Moresby (G. Mola);; University of New South Wales, Sydney, New South Wales, Australia (A. Rosewell, A. Zwi, C.R. MacIntyre)

**Keywords:** cholera, emergence, outbreak, bacteria, Vibrio cholerae O1, New Guinea, letter

**To the Editor:** Cholera outbreak reports are of international public health interest, especially in areas that were previously cholera free (*1*). Although many recent cholera outbreaks have originated in coastal areas (*2*), identifying the source of cholera introduction has been challenging (*1*). The detection of *Vibrio* c*holerae* in coastal, brackish and riverine waters in cholera-endemic and cholera-free areas supports the view that autochtonous *V. cholerae* is involved in the introduction of cholera (*3**,**4*). To our knowledge, cholera has not been reported in Papua New Guinea, despite social and environmental conditions likely to facilitate transmission and the nation's close proximity to cholera-endemic countries (*5**,**6*).

On August 6, 2009, a physician who visited the coastal village of Lambutina reported an outbreak of acute watery diarrhea that was associated with the death of his father and 4 other persons from this and a neighboring village. The outbreak began in the village of Nambariwa and spread to neighboring Lambutina, Morobe Province. From August 13, multidisciplinary teams worked with the community to reduce the number of deaths through early identification and treatment of case-patients. The teams also worked to limit transmission through improvements to the water and sanitation infrastructure and by encouraging better hygiene practices among the villagers. A suspected case of cholera was defined as acute watery diarrhea or vomiting in a resident of Lambutina or Nambariwa villages since July 22, 2009. In the 2 villages, 77 cases were identified; attack rates were 14% in Lambutina (48/343) and 5.5% in Nambariwa (29/532). The overall case-fatality ratio was 6.5% (5/77); 2 patients died after they were discharged from the referral hospital.

A retrospective frequency-matched case–control study was conducted in Lambutina to identify the risk factors associated with suspected cholera. Neighborhood controls (± 5 years of age) were selected from unaffected households. Univariate and multivariate analyses were conducted with STATA version 10 (StataCorp., College Station, TX, USA).

Of the 48 case-patients in Lambutina, 43 participated in the study with 43 age-matched controls. In addition to having close contact with patients who had cholera, univariate analysis showed that case-patients were more likely to have had several exposures related to the death of other patients (Table). However, having close contact with a patient was the only independent risk factor (adjusted odds ratio 4.8, 95% confidence interval 1.7–13.4) (Table). Close contact included providing nursing care for patients or carrying patients onto boats for transport to health care facilities.

From the 10 collected samples, 4 isolates were confirmed as *V. cholerae* O1, biotype El Tor, serotype Ogawa, by PCR detection of an O1-specific region of the *rfb* gene using established methods and PCR amplification of the *tcpA* gene polymorphism specific for the El Tor biotype (*7*). The *ctx*AB, *vct* genes (present in toxigenic strains) and the hemolysin gene *hly*A (present in all *V. cholerae* strains) were detected by PCR in all 4 isolates.

Although health authorities promptly identified and responded to the outbreak, they could not determine its origin. The El Niño weather phenomenon generates increased rainfall and elevated sea surface temperatures and is a predictor of cholera outbreaks (*8*), which puts more coastal areas at risk for such outbreaks (*9*). During this outbreak, Papua New Guinea reported above-average rainfall (*10*) and warmer sea surface temperatures. Although cholera may have been introduced to Papua New Guinea through an infectious traveler or by other means, climatic factors may have initiated plankton blooms, the abundance of which have also been associated with increased presence of *V. cholerae* O1. Sea and estuarine waters of these villages are plausible sources of introduction.

In Lambutina, the age-specific attack rates were lowest among young children and increased among persons of middle age and among the elderly. Those providing patient care and lifting during transportation as well as those washing the bodies of the deceased may have been more represented in the >40 years age group; however, this situation may not explain the high attack rates among the elderly.

Generally, after a cholera outbreak is detected, interventions aim to reduce the proportion of deaths to <1%. The overall case-fatality ratio in the outbreak discussed here was 6.5%, which reflects the challenges to accessing adequate health care in remote settings. This difficulty is exacerbated when the disease occurs for the first time because cholera awareness and preparedness will be weak, as can be seen in the early management of cases during this outbreak. Villagers who have close contact with cholera patients are at greater risk for disease and should be a focus of interventions to limit transmission (e.g., eliminating ingestion of contaminated water, improving hygiene and sanitation). Education to increase awareness of the disease and enhanced access to low-osmolarity oral rehydration solution, Hartmann solution, and zinc supplements are essential.

Cholera-endemic and cholera–nonendemic countries with coastal populations are at an increasing risk for cholera outbreaks. Adequate preparation by the health care system is vital to avoid excess deaths.

**Table:** Univariate and multivariate analysis of risk factors associated with suspected cholera in Lambutina village, Papua New Guinea, 2009*

Risk factor	Univariate analysis					Multivariate analysis	
	No. cases (%), n = 43	No. controls (%), n = 43	OR (95% CI)	p value		aOR (95% CI)	p value
Attended a funeral	32 (74)	24 (56)	2.3 (0.8–6.4)	0.07		1.8 (0.7–4.9)	0.25
Had death in the family	8 (19)	1 (2)	9.6 (1.1–214.6)	0.02		2.6 (0.2–43.9)	0.51
Consumed food during funeral	38 (88)	34 (79)	2.0 (0.5–7.8)	0.24		NA	NA
Washed the body/clothes of deceased	7 (16)	1 (2)	8.2 (0.9–185.1)	0.03		1.6 (0.1–28.1)	0.74
Had close contact with diarrhea patient	25 (58)	8 (19)	6.1 (2.1–18.3)	0.001		4.8 (1.7–13.4)	0.003
Drank tap water	43 (100)	43 (100)	1.0 (NA)	NA		NA	NA
Boiled water for consumption	1 (2)	0	1.0 (NA)	NA		NA	NA
Washed utensils in the ocean	39 (91)	39 (91)	1.0 (0.2–5.2)	0.64		NA	NA

*OR, odds ratio; CI, confidence interval; aOR, adjusted OR; NA, not applicable.
